# Impact of the COVID-19 pandemic on the mental health of nursing students in Japan: a cross-sectional study

**DOI:** 10.1265/ehpm.22-00128

**Published:** 2022-10-15

**Authors:** Yoshiyasu Ito, Jun Kako, Kohei Kajiwara, Yasutaka Kimura, Takahiro Kakeda, Seiji Hamanishi, Shinsuke Sasaki, Makoto Yamanaka, Hana Kiyohara, Yuki Wakiguchi, Yoji Endo, Kimie Harada, Yuji Koga, Michiko Ishida, Yoko Nishida, Masamitsu Kobayashi, Michihiro Tsubaki

**Affiliations:** 1 College of Nursing Art and Science, University of Hyogo, Akashi 673-8588, Japan; 2 Graduate School of Health Sciences, Kobe University, Kobe 654-0142, Japan; 3 Faculty of Nursing, Japanese Red Cross Kyushu International College of Nursing, Munakata 811-4157, Japan; 4 Department of Nursing, Meio University, Nago 905-8585, Japan; 5 Kawasaki City College of Nursing, Kawasaki 212-0054, Japan; 6 Faculty of Nursing, Kansai University of Social Welfare, Ako 678-0255, Japan; 7 Department of Nursing Science, Okayama Prefectural University, Soja 719-1197, Japan; 8 School of Nursing, Aichi Medical University, Nagakute 480-1195, Japan; 9 Faculty of Nursing, Kawasaki University of Medical Welfare, Kurashiki 701-0193, Japan; 10 Graduate of Nursing Science, St. Luke’s International University, Tokyo 104-0044, Japan; 11 School of Nursing, Kitasato University, Sagamihara 252-0329, Japan

**Keywords:** Anxiety, COVID-19, Depression, Insomnia, Nursing student

## Abstract

**Background:**

The effect of the prolonged coronavirus disease (COVID-19) pandemic on the mental health of nursing students is unclear. This study assesses the prevalence of anxiety, depression, and insomnia among nursing students in Japan during the pandemic and determines the risk factors associated with such symptoms.

**Methods:**

An online survey-based cross-sectional study was conducted from August 16 to October 16, 2021. Participants were first- to fourth-year nursing students enrolled in undergraduate programs at the eight universities in Japan. Anxiety, depression, and insomnia were assessed using the Generalized Anxiety Disorder-7, Patient Health Questionnaire-9, and Insomnia Severity Index-7, respectively. We calculated descriptive statistics for each measurement item and performed univariate and logistic regression analyses to evaluate the potential risk factors.

**Results:**

We received responses from 1,222 of 3,056 nursing students (response rate: 40.0%). After 25 participants were excluded due to missing outcome values, 1,197 students (valid response rate: 98.0%) were included in the analysis. The prevalence of anxiety, depression, and insomnia was 4.8%, 12.4%, and 18.0%, respectively. The risk of anxiety was lower among participants who did not have any relatives or friends who had been infected with SARS-CoV-2 than among those who did (aOR 0.36, 95% CI 0.14–0.94). The risk of depression was higher among participants whose financial status had worsened during the pandemic than among those whose financial status had not changed (aOR 3.44; 95% CI 1.98–5.96). Common factors that increased the risk of anxiety, depression, and insomnia were life satisfaction and fear of COVID-19.

**Conclusion:**

Mental health-related symptoms among nursing students in Japan have not necessarily worsened with the spread of COVID-19 but were exacerbated by the intensity of changes in daily living and fear, which are psychosocial effects associated with the pandemic.

**Supplementary information:**

The online version contains supplementary material available at https://doi.org/10.1265/ehpm.22-00128.

## Introduction

The novel coronavirus disease (COVID-19) pandemic began in Wuhan, China, in December 2019, and subsequently spread rapidly, threatening global health [1]. As of January 31, 2022, there were 373,229,380 confirmed cases worldwide, with 5,658,702 deaths [2]. In Japan, since the first case of COVID-19 was confirmed in January 2020, 2,730,828 cases and 18,792 deaths have been estimated [3].

The state of the pandemic varies among countries due to differences in COVID-19 responses by respective governments, systems for maintaining healthcare, and demographics (i.e., the aging rate). In Japan, in comparison to other countries with similar total numbers of cases, it is characterized by a low mortality rate [2]. The state of emergency declaration by the Japanese government in dealing with COVID-19 is a voluntary lockdown [4], which rates lower than other developed nations on a numerical index of the severity of COVID-19 responses enacted by governments of various countries [5, 6].

COVID-19 is known to affect not only physical health but also mental health [7], and, the prevalence of anxiety, depression, and insomnia among the general population during the pandemic is reported to be 31.9%, 31.4%, and 37.9%, respectively [8]. Among healthcare workers, the reported prevalence of these respective symptoms is 23.2%, 22.8%, and 38.9%, respectively [9]. The tremendous burden that the COVID-19 pandemic has placed on healthcare workers and the entire medical system [10], and its serious effects on the mental health of healthcare workers have led to the collapse of the health care system, thus posing a major public health problem.

The adverse impact of the COVID-19 pandemic on the mental health of nurses is due to factors such as performing their major responsibility in the hospitals despite exposing themselves to the risk of infection and discrimination from others [11]. With the progression of the pandemic, several nurses have quit their jobs, thus leading to a shortage of nurses [12]. Similar to previous pandemics, nurses are more likely to quit their jobs compared to that by people in other professions [13, 14]. The pandemic has affected the mental health of not only nurses but also nursing students; as COVID-19 anxiety has increased, nursing students currently have a negative mindset about the profession [15]. The demand for healthcare workers is predicted to increase with the progression of the COVID-19 pandemic [16–18]. Therefore, maintaining the healthcare system urgently requires focusing on the mental health of not only those working as nurses currently but also of the nursing students who will be future nurses and providing them with appropriate support.

Studies on the mental health-related symptoms of nursing students during the COVID-19 pandemic have examined anxiety, depression, insomnia, stress, and fear. A meta-analysis found the prevalence of these symptoms to be 32.0%, 52.0%, 27.0%, 30.0%, and 41%, respectively [19]. These rates are higher than those for the general population [8] and for healthcare workers [9]. Conversely, a recent study found the prevalence of anxiety and depression among nursing students to be 2.9% [20], which is extremely lower than that found in previous studies. Most relevant studies were conducted in 2020, and findings on mental health related symptoms among nursing students reflecting the current status of COVID-19 have not been implemented.

Furthermore, although various psychosocial factors such as COVID-19-associated changes in financial status and daily living, life satisfaction, and fear of the pandemic may affect mental health-related symptoms among nursing students, no previous studies have examined these factors.

This study assesses the mental health, including anxiety, depression, and insomnia, of nursing students in Japan during the COVID-19 pandemic and determines the latent risk factors associated with these symptoms. The results can serve as important evidence for providing nursing students with appropriate mental health support during the pandemic.

## Methods

### Study design

This study is a cross-sectional study that recruited nursing students enrolled in Japanese universities. It was approved by the Institutional Review Board of the College of Nursing Art and Science, University of Hyogo (approval no.: 2020F29). Clinical trial registration for this study was completed before it was begun (University Hospital Medical Information Network, Japan [UMIN000044355]), and the protocol for the study is reported in detail elsewhere [21]. The results of this study are reported in accordance with Strengthening the Reporting of Observational Studies in Epidemiology guidelines [22].

### Setting

The nursing students were recruited from the following eight universities in different geographical regions (Kanto, Kansai, Chugoku, Kyushu, and Okinawa) of Japan: University of Hyogo, Kansai University of School Welfare, Japanese Red Cross Kyushu International College of Nursing, Kawasaki University of Medical Welfare, Okayama Prefecture University, Meio University, Kitasato University, and Aichi Medical University.

The data for this study were collected from August 16, 2021 to October 16, 2021 via online surveys conducted using Microsoft Forms (Microsoft Office 365, USA). The study was conducted in accordance with the previously established protocol [21].

### Participants

The participants were nursing students enrolled in undergraduate programs at the eight selected universities. Eligibility criteria consisted of the following: providing consent to participate in this study, being enrolled in a department of nursing at a four-year university, and being a first-year to fourth-year student at one of the eight participating universities.

### Outcome measures

The Japanese versions of the following three measures were used to assess the mental health of the participants [23–25].

Anxiety was assessed using the Generalized Anxiety Disorder-7 (GAD-7) questionnaire [23]. It is a seven-item questionnaire that is widely used to screen for generalized anxiety disorder. Total scores range from 0 to 21 and are classified into the following four categories: no anxiety (0–4), mild anxiety (5–9), moderate anxiety (10–14), and severe anxiety (15–21). The Japanese version of the GAD-7 questionnaire has been confirmed to be valid and useful [26, 27].

Depression was assessed using the Patient Health Questionnaire-9 (PHQ-9) [24]. The PHQ-9 is a nine-item questionnaire that is widely used to screen for major depression. Total scores range from 0 to 27 and are classified into the following five categories: no depression (0–4), mild depression (5–9), moderate depression (10–14), moderately severe depression (15–19), and severe depression (20–27). The Japanese version of the PHQ-9 has been confirmed to be valid and useful [28, 29].

Insomnia was assessed using the Insomnia Severity Index-7 (ISI-7) [25]. The ISI-7 comprises seven items regarding insomnia over the past two years. Total scores range from 0 to 28 and are classified into the following four categories: absence of insomnia (0–7), sub-threshold insomnia (8–14), moderate insomnia (15–21), and severe insomnia (22–28). The Japanese version of the ISI-7 has been reported to be valid and reliable [30].

Furthermore, to assess latent risk factors for the mental health of nursing students in a multifaceted fashion, we considered risk factors associated with the psychosocial effects of the COVID-19 pandemic. Therefore, in addition to demographic characteristics, we obtained data on the following variables: economic deprivation since the COVID-19 pandemic began, physical activities, life satisfaction, and sense of fear. Economic deprivation was assessed on the following five-point scale: “My financial situation became difficult,” “My financial situation became a little difficult,” “My financial situation remained the same,” “My financial situation became a bit comfortable,” and “My financial situation became comfortable.”

Physical activity was assessed, using the Japanese version of the International Physical Activity Questionnaire-Short Form (IPAQ-SF) [31], as “low,” “moderate,” or “high.” The Japanese version of the IPAQ-SF has been confirmed to be highly reliable and valid [32]. Change in physical activity since the pandemic began was classified into the following four categories: “no exercise,” “decreased,” “unchanged,” and “increased.”

Based on a study by Tang et al. [33], the following two parameters of life satisfaction were assessed: “life satisfaction” and “change in life satisfaction since the start of the pandemic.” Life satisfaction was assessed on an 11-point Likert scale from “extremely unsatisfied” to “extremely satisfied.” Change in life satisfaction since the start of the pandemic was assessed on the following five-point scale: “a lot worse,” “worse,” “pretty much the same,” “better,” and “a lot better.”

Sense of fear was assessed in terms of fear of COVID-19 using the Fear of Coronavirus-19 Scale (FCV-19S) [34]. The FCV-19S is a seven-item scale that quantitatively measures the fear of COVID-19. Total scores range from 7 to 35, with a higher score representing greater fear of COVID-19. The validity of the FCV-19S as a measure of fear of COVID-19 has been examined in several countries [35–39]; the Japanese version of the FCV-19S has been reported to be highly valid and reliable [40]. The FCV-19S has also been confirmed to be valid and reliable as a scale for measuring the fear of COVID-19 among university students [35].

Demographic characteristics included sex, year in school, body mass index (BMI), living with family members, SARS-CoV-2 infection status of relatives and acquaintances, smoking status, and change in alcohol consumption. BMI was calculated based on the height and body weight of participants and was classified into the following three categories: underweight (BMI < 18.5 kg/m^2^), normal range (18.5–24.9 kg/m^2^), and obese (≥25.0 kg/m^2^). A BMI of ≥25.0 kg/m^2^ was defined as obese in accordance with the Japanese obesity examination guidelines. Change in alcohol consumption was assessed using the following four-point scale: “no alcohol consumption,” “decreased,” “unchanged,” and “increased.”

### Sample size

The target sample size was determined based on population proportion interval estimation. The proportion of nursing students with mental health issues was estimated to be roughly 30% in previous studies [41, 42]. With this proportion, the sample size with an error (δ) of 2.5% and a reliability (1 − α) of 95% is 1,291; therefore, we targeted to obtain survey responses from 1,300 participants. The sample size was calculated using EZR statistical software (Saitama Medical Centre, Jichi Medical University, Saitama, Japan) and R (The R Foundation for Statistical Computing, Vienna, Austria) [43]. With a presumed survey response rate of approximately 40%, the survey was administered to approximately 3,000 participants.

### Statistical analysis

After excluding missing values in the GAD-7, PHQ-9, and ISI-7 (the outcome measures), we included all other data in the analyses.

We first calculated descriptive statistics for all variables. We assessed the severities of anxiety, depression, and insomnia (the outcomes) based on total scores for the GAD-7, PHQ-9, and ISI-7, respectively, and represented the distribution of severities in terms of frequency and proportion.

The cutoff score for detecting anxiety, depression, and insomnia was 10 [23–25]. Regarding the cutoff point of ISI-7 in Japanese, the area under the receiver operating characteristic (ROC) curve was largest when the cutoff score was 9.5 points (AUC, 0.93; 95% CI, 0.89–0.97). Sensitivity and specificity were 88.7% and 85.8%, respectively, when the cutoff score was 10 points or higher, indicating that a cutoff score of 10 points is appropriate for use with the Japanese version of this scale [26]. Participants with a score of ≥10 were considered as having the symptom in question; the percentages of participants with each symptom are represented as the prevalence. Categorical variables are presented as frequency and proportion while continuous variables with a normal distribution are presented as mean and standard deviation and with a non-normal distribution are presented as median and interquartile range (IQR).

To confirm correlations among anxiety, depression, and insomnia, we calculated Pearson’s correlation coefficients among total scores for the GAD-7, PHQ-9, and ISI-7.

To assess latent risk factors for anxiety, depression, and insomnia, we performed multiple logistic regression analysis with dichotomous GAD-7, PHQ-9, and ISI-7 scores (below cutoff versus cutoff or higher) as dependent variables; associations with risk factors are presented with odds ratios (OR) and 95% confidence intervals (CI). All variables associated with GAD-7, PHQ-9, and ISI-7 dichotomous scores with P < .200 in a univariate analysis were entered into the multiple logistic regression analysis as explanatory variables via forced entry. The significance level was set at P < .050. Regarding variables forced into the regression model, we confirmed the absence of multicollinearity based on the variance inflation factor (VIF); a VIF ≥ 5.0 was considered to represent multicollinearity [44]. All analyses were conducted with SPSS version 26 (SPSS Inc., Chicago, IL).

### Patient and public involvement

Patients and the public were not involved in any way in this research.

## Results

### Characteristics of participants

Of the 3,056 nursing students included in this study, 1,222 responded to the survey (response rate, 40.0%). After excluding 25 of these students due to missing values for the GAD-7, PHQ-9, or ISI-7, we included the remaining 1,197 students in the analyses (valid response rate, 98.0%). Of these students, 1,126 were women (94.1%). There were more first-year students (n = 322, 26.9%) than students in any other year. A total of 700 students (58.5%) lived with their families, and 248 students (20.7%) had a relative or friend who had been infected with SARS-CoV-2. Descriptive statistics for the complete characteristics of participants are reported in Table 1.

**Table 1:** Characteristics of Participants

**Characteristic**	**No. (%)^a^**
**Sex**	
Men	71 (5.9)
Women	1126 (94.1)
**Grade in school**	
First	322 (26.9)
Second	292 (24.4)
Third	282 (23.6)
Fourth	301 (25.1)
**BMI**	
Underweight (<18.5 kg/m^2^)	236 (20.3)
Normal range (18.5–24.9 kg/m^2^)	890 (76.7)
Obese (≧25.0 kg/m^2^)	35 (3.0)
**Living with family**	
No	497 (41.5)
Yes	700 (58.5)
**Relatives or friends who have been infected with SARS-CoV-2**	
No	949 (79.3)
Yes	248 (20.7)
**Smoking status**	
No	1077 (90.4)
Yes	114 (9.6)
**Change in alcohol consumption since start of pandemic**	
No alcohol consumption	664 (55.7)
Decreased	239 (20.0)
Unchanged	230 (19.3)
Increased	60 (5.0)

Abbreviations: BMI, Body Mass Index; COVID-19, Coronavirus Disease 2019

^a^Descriptive statistics are based on data containing missing values, and the total number of participants included in the analysis may be less than 1,197.

### Impact of the COVID-19 pandemic on daily life and sense of fear

A total of 117 students (9.8%) reported that their financial situation had become difficult since the start of the pandemic, while 342 students (28.6%) stated that their financial situation had become slightly difficult. For 715 students (60.0%), physical activity had decreased since the start of the pandemic. Regarding change in life satisfaction since the start of the pandemic, 156 students (13.0%) responded with “a lot worse,” while 720 responded with (60.2%) “worse.” The median (IQR) for life satisfaction was 5.0 (3.0–7.0). The mean FCV-19S score for fear of COVID-19 was 17.26 ± 5.09 (Table 2).

**Table 2:** Impact of the COVID-19 Pandemic on Daily Life and Sense of Fear

**Characteristic**	**No. (%)^a^**
**Change in financial situation since start of pandemic**	
Became difficult	117 (9.8)
Became a little difficult	342 (28.6)
Remained the same	651 (54.5)
Became a bit comfortable	75 (6.3)
Became comfortable	10 (0.8)
**Physical activity level^b^**	
Low	638 (53.3)
Moderate	407 (34.0)
High	152 (12.7)
**Change in physical activity level since start of pandemic**	
No exercise	111 (9.3)
Decreased	715 (60.0)
Unchanged	280 (23.5)
Increased	86 (7.2)
**Life satisfaction^c^**	
Median (IQR)	5.0 (3.0–7.0)
**Change in life satisfaction since start of pandemic**	
A lot worse	156 (13.0)
Worse	720 (60.2)
Pretty much the same	253 (21.1)
Better	62 (5.2)
A lot better	6 (0.5)
**Fear of COVID-19^d^**	
Mean ± SD	17.26 ± 5.09

Abbreviations: BMI, Body Mass Index; COVID-19, Coronavirus Disease 2019; IQR, Interquartile Range; SD, Standard Deviation

^a^Descriptive statistics are based on data containing missing values, and the total number of participants included in the analysis may be less than 1,197.

^b^Current level of physical activity was assessed using the International Physical Activity Questionnaire-Short Form

^c^Current life satisfaction was assessed with an 11-point Likert scale ranging from “extremely unsatisfied” to “extremely satisfied”

^d^Fear of COVID-19 was assessed with the Fear of Coronavirus-19 Scale

### Mental health outcomes

The prevalence of anxiety, depression, and insomnia was 4.8%, 12.4%, and 18.0%, respectively. The classification of each symptom by severity is shown in Table 3. Pearson correlation coefficients among the GAD-7, PHQ-9, and ISI-7 were as follows: between the GAD-7 and PHQ-9, r = .696 (p < .001); between the GAD-7 and the ISI-7, r = .469 (p < .001); and between the PHQ-9 and ISI-7, r = .666 (p < .001).

**Table 3:** Severity Categories of Anxiety, Depression, and Insomnia Symptoms

**Severity categories**	**No. (%)**
**GAD-7, Anxiety Symptoms**	
Normal	909 (75.9)
Mild	230 (19.2)
Moderate	47 (3.9)
Severe	11 (0.9)
**PHQ-9, Depression Symptoms**	
Normal	724 (60.5)
Mild	325 (27.2)
Moderate	99 (8.3)
Moderately severe	35 (2.9)
Severe	14 (1.2)
**ISI-7, Insomnia Symptoms**	
Absence	848 (70.8)
Subthreshold	279 (23.3)
Moderate	63 (5.3)
Severe	7 (0.6)

Abbreviations: GAD-7, Generalized Anxiety Disorder-7; PHQ-9, Patient Health Questionnaire-9; ISI-7, Insomnia Severity Index-7

### Risk factors for mental health outcomes

In the univariate analysis, the following variables were associated with at least one symptom (anxiety, depression, or insomnia): sex, BMI, living with family, having a relative or friend who had been infected with SARS-CoV-2, change in financial situation since the start of the pandemic, change in physical activity since the start of the pandemic, life satisfaction, change in life satisfaction since the start of the pandemic, and fear of COVID-19 (Additional file 1). The VIF among the variables given above ranged from 1.018 to 3.224; therefore, all variables were forced into the multiple logistic regression analysis as explanatory variables. The results from the multiple logistic regression analysis showed that participants with a relative or friend who had been infected with SARS-CoV-2 were at lower risk for anxiety than those without a relative or friend who had been infected with SARS-CoV-2 (aOR 0.36, 95% CI 0.14–0.94). Compared to participants with normal BMI, those who were obese were at higher risk of depression and insomnia (depression: aOR 3.30, 95% CI 1.42–7.69; insomnia: aOR 2.83, 95% CI 1.34–5.98). The participants who stated that their financial situations had become difficult since the start of the pandemic were at higher risk of depression than that observed with participants whose financial situations had not changed (aOR 3.44, 95% CI 1.98–5.96), while participants whose financial situations had become a little difficult were at higher risk of insomnia (aOR 1.44, 95% CI 1.01–2.05) than that observed with participants whose financial situation had not changed. The risk factors common to increased risk of anxiety, depression, and insomnia were life satisfaction and fear of COVID-19. Detailed results for the multiple logistic regression analysis are shown in Table 4.

**Table 4:** Risk Factors for Anxiety, Depression, and Insomnia Symptoms by Multivariable Logistic Regression Analysis

**Variable**	**GAD-7, Anxiety Symptoms**		**PHQ-9, Depression Symptoms**		**ISI-7, Insomnia Symptoms**	
		
	**aOR**	**95% CI**	**aOR**	**95% CI**	**aOR**	**95% CI**
**Sex**						
Men	Reference					
Women	0.69	0.22–2.13	1.49	0.63–3.52	0.77	0.41–1.45
**BMI**						
Normal range (18.5–24.9 kg/m2)	Reference					
Underweight (<18.5 kg/m2)	0.97	0.46–2.09	1.45	0.91–2.29	**1.61**	**1.11**–**2.35**
Obese (≧25.0 kg/m2)	1.94	0.60–6.26	**3.30**	**1.42**–**7.69**	**2.83**	**1.34**–**5.98**
**Living with family**						
No	Reference					
Yes	1.71	0.92–3.16	0.99	0.67–1.45	0.86	0.63–1.19
**Relatives or friends who have been infected with SARS-CoV-2**						
No	Reference					
Yes	**0.36**	**0.14**–**0.94**	0.62	0.37–1.04	0.93	0.63–1.40
**Change in financial situation since start of pandemic**						
Unchanged	Reference					
Became comfortable or became a bit comfortable	1.14	0.32–4.06	1.63	0.74–3.59	0.88	0.42–1.82
Became a little difficult	1.34	0.71–2.55	1.51	0.98–2.35	**1.44**	**1.01**–**2.05**
Became difficult	1.86	0.80–4.30	**3.44**	**1.98**–**5.96**	1.45	0.86–2.44
**Change in physical activity level since start of pandemic**						
Unchanged or no exercise	Reference					
Decreased	0.68	0.37–1.25	1.51	0.97–2.35	1.26	0.88–1.81
Increased	0.22	0.03–1.69	0.85	0.33–2.17	0.76	0.36–1.61

**Life satisfaction^b^**	**0.77**	**0.64**–**0.93**	**0.80**	**0.71**–**0.91**	**0.88**	**0.79**–**0.97**

**Change in life satisfaction since start of pandemic**						
Pretty much the same	Reference					
A lot better or better	0.54	0.07–4.54	0.79	0.25–2.49	1.28	0.58–2.83
Worse	0.51	0.21–1.20	0.60	0.33–1.07	0.66	0.41–1.05
A lot worse	0.91	0.30–2.74	0.99	0.46–2.14	1.37	0.72–2.61

**Fear of COVID-19^c^**	**1.10**	**1.04**–**1.16**	**1.06**	**1.02**–**1.10**	**1.05**	**1.01**–**1.08**

Abbreviations: aOR, adjusted Odds ratio; BMI, Body Mass Index; CI, confidence interval; COVID-19, Coronavirus Disease 2019; GAD-7, Generalized Anxiety Disorder-7; PHQ-9, Patient Health Questionnaire-9; ISI-7, Insomnia Severity Index-7

^b^Current life satisfaction was assessed with an 11-point Likert scale ranging from “extremely unsatisfied” to “extremely satisfied”

^c^Fear of COVID-19 was assessed with the Fear of Coronavirus-19 Scale

## Discussion

To our knowledge, this study is the first to determine the most recent distributions of anxiety, depression, and insomnia among nursing students during the COVID-19 pandemic and to report the risk factors for those symptoms based on a multifaceted assessment that includes psychosocial factors.

We found that although the data for this study was obtained when the numbers of newly confirmed COVID-19 cases in Japan were at the highest since the pandemic begun (see Additional file 2), the prevalence of anxiety, depression, and insomnia among nursing students was lower than that in previous studies. A systematic review and meta-analysis of the prevalence of anxiety, depression, and insomnia in nursing students reported prevalence rates of 32.0%, 52.0%, and 27.0%, respectively [45]. However, in our study, we found that the prevalence of anxiety, depression, and insomnia was 4.8%, 12.4%, and 18.0%, respectively. This difference may be explained by the fact that at the time of the study, the number of coronavirus (COVID-19) vaccinations was increasing and COVID-19 related fatalities were decreasing significantly in Japan. Most previous studies were based on data from 2020 [45], when the COVID-19 vaccine had not yet become widely available. There were extremely few COVID-19 vaccinations in each country in the same period in 2020; in the US, which was the country with the highest vaccine coverage at that time, only 0.1% of the population was vaccinated by December 31 [46]. In contrast, in our study, data collection began on August 16, 2021, at which point 41.7% of the population in Japan had been fully immunized. This increased to 70.1% by October 16, 2021, when data collection was completed; that is, the percentage of people who had been fully immunized was high [46]. Additionally, the moving-average case fatality rate of COVID-19 in Japan rose to a peak of 5.03% on March 8, 2021 [47] and subsequently decreased, partly because of the increasing availability of vaccines. During the data collection period for our study, the moving-average case fatality rate of COVID-19 further decreased to 0.16–2.03%, which was low compared to that reported in other countries [47]. An investigation of the association between COVID-19 vaccination and mental health demonstrated that vaccination was associated with reduced mental anguish, perceived risk of infection, hospitalization, and death, and improved mental health [48]. In addition, in our study, the fear of COVID-19 among nursing students was lower than that reported in previous studies [49, 50]. These results suggest that the low prevalence of mental health-related symptoms among nursing students in our study could be due to a reduction in perceived risk of infection and danger of COVID-19 associated with the increasing availability of COVID-19 vaccines and decreased fatality rate.

The effects of the COVID-19 pandemic on daily life and studies are associated with anxiety among nursing students [42]. Changes in daily life wrought by the pandemic have been demonstrated to worsen mental health. However, regarding the long-term effects of these pandemic-associated changes in daily life, while deterioration of mental health was observed immediately after lockdowns were implemented, levels of anxiety and depression subsequently decreased rapidly, thus demonstrating that people have mentally adapted to changes in daily life [51, 52]. Nursing students have are known to be particularly adept at coping during the COVID-19 pandemic, and many consider themselves as sufficiently resilient [53]. In Japan, the third COVID-19-related state of emergency was declared on April 23, 2021 and continued for approximately five more months until September 28, 2021. During that long period, nursing students mentally adapted to the changes in daily life, thus leading to a low prevalence of mental health-related issues.

We found that a worsening financial situation, increased fear of COVID-19, and decreased life satisfaction are latent risk factors that stem from the psychosocial effects of the pandemic on the mental health of nursing students. Previous studies reported that a worsening financial situation is a risk factor for anxiety and depression in nursing students during a pandemic [54, 55] and fear of COVID-19 is a risk factor for anxiety [15]. Our study replicated these findings, but additionally found for the first time that a worsening financial situation is a risk factor for insomnia symptoms and fear of COVID-19 is a risk factor for depression and insomnia. Furthermore, life satisfaction is a risk factor for anxiety and depression among university students [56]; our study found that life satisfaction is also a risk factor for anxiety and depression among nursing students, in addition to being a risk factor for insomnia.

In contrast, our study demonstrated that the risk of anxiety was lower among nursing students who had relatives or friends infected with SARS-CoV-2 than among students with no infected relative or friend. This result differs from that of a previous study that reported that knowing someone who had had COVID-19 was a risk factor for observing the psychosocial effects of COVID-19 among university students [57]; this divergence in results is conceivably due to reductions in serious illness and mortality among patients with COVID-19. Many nursing students live with the fear and anxiety of infecting family members and other people close to them with SARS-CoV-2 [53]; thus, nursing students’ anxiety may be attributed to the fear that they could be infected or they could infect their family [42]. Note that during the data collection period of this study, the fatality rate associated with COVID-19 decreased significantly in Japan and was lower than that reported in other countries. Thus, the nursing students’ fear and worry of infecting someone close to them may have reduced in our study because none of the people close to them who had COVID-19 developed serious illness or died. Our study suggested that the mental health of nursing students during the COVID-19 pandemic does not necessarily deteriorate despite the increasing numbers of cases and infections among people close to the students but instead deteriorates due to the intensity of psychosocial factors, namely pandemic-related changes in daily life and fear of COVID-19. Therefore, the effects of the COVID-19 pandemic on the mental health of nursing students cannot be predicted based on only the changes in the number of cases; instead, it is necessary to monitor changes in daily life and perceptions of COVID-19 and to provide psychosocial support tailored to address these changes.

### Limitations

This study has several limitations. First, we did not collect data regarding the COVID-19 vaccination status of the participants at the time of the survey. The absence of this data limits interpretations of the association between COVID-19 vaccination and mental health. Second, because the prevalence of anxiety, depression, and insomnia among the participants in this study was lower than that assumed, the sample size may have been insufficient, thereby reducing the accuracy of our analysis. Third, this study is an observational study, which limits interpretations of causal relationships. In particular, life satisfaction may have decreased due to restrictions on daily activities imposed by anxiety, depression, and insomnia. Finally, the survey response rate was only 40.0%, which indicates the possibility of selection bias; for example, non-respondents may have been unable to respond due to mental health-related symptoms or may have had no interest in this study due to a complete absence of symptoms. However, our study recruited participants from geographically different regions throughout Japan, which makes the data relatively representative of the entire country. We also assessed mental health comprehensively by including psychosocial factors, namely changes in daily living and fear of COVID-19.

## Conclusion

Our study is the first to determine the distribution of anxiety, depression, and insomnia among nursing students in Japan during the COVID-19 pandemic. We found that despite the increasing spread of COVID-19, the prevalence of these symptoms was lower than that in previous studies. We also found that worsened financial situation, life satisfaction, and fear of COVID-19 are latent risk factors that stem from the psychosocial effects of the pandemic for anxiety, depression, and insomnia among nursing students. The mental health of nursing students during the COVID-19 pandemic is not necessarily worsened by an increase in the number of cases or a relative or friend being infected; rather, our results suggested that the mental health of nursing students is exacerbated by the intensity of the psychosocial effects of the pandemic such as changes in daily life and fear of the disease.

## Data Availability

Data are available upon reasonable request.

## Supplementary information

**Additional file 1:** Factors Associated with Anxiety, Depression, and Insomnia Symptoms by Univariate Analysis.

**Additional file 2:** Coronavirus Disease 2019 (COVID-19) pandemic in Japan^a^.
