# Supplementary material for: Impact of the COVID-19 pandemic on the mental health of nursing students in Japan: a cross-sectional study
Source: Environ Health Prev Med. 2022 Oct 15;27:40. doi: 10.1265/ehpm.22-00128 (PMC9640737; doi:10.1265/ehpm.22-00128)
Supplement: Supplementary file 1 — Additional file 1: Factors Associated with Anxiety, Depression, and Insomnia Symptoms by Univariate Analysis. [file ehpm-27-040-s001.pdf]

Additional File 1. Factors Associated with Anxiety, Depression, and Insomnia Symptoms by Univariate Analysis

Variable	GAD-7, Anxiety Symptoms			PHQ-9, Depression Symptoms			ISI-7, Insomnia Symptoms		
	No. (%)			No. (%)			No. (%)		
	GAD-7 ≤9	GAD-7 ≥10	P-value [a]	PHQ-9 ≤9	PHQ-9 ≥10	P-value [a]	ISI-7 ≤9	ISI-7 ≥10	P-value [a]
**Sex**									
Men	67 (94.4)	4 (5.6)	.772	63 (88.7)	8 (11.3)	.772	54 (76.1)	17 (23.9)	.183
Women	1072 (95.2)	54 (4.8)		986 (87.6)	140 (12.4)		927 (82.3)	199 (17.7)	
**Year in school**									
First	310 (96.3)	12 (3.7)	.304	282 (87.6)	40 (12.4)	.369	266 (82.6)	56 (17.4)	.351
Second	272 (93.2)	20 (6.8)		251 (86.0)	41 (14.0)		243 (83.2)	49 (16.8)	
Third	269 (95.4)	13 (4.6)		244 (86.5)	38 (13.5)		221 (78.4)	61 (21.6)	
Fourth	288 (95.7)	13 (4.3)		272 (90.4)	29 (9.6)		251 (83.4)	50 (16.6)	
**BMI**									
Underweight （<18.5 kg/m2）	227 (96.2)	9 (3.8)	.150	206 (87.3)	30 (12.7)	.012	187 (79.2)	49 (20.8)	.005
Normal range (18.5–24.9 kg/m2)	846 (95.1)	44 (4.9)		786 (88.3)	104 (11.7)		741 (83.3)	149 (16.7)	
Obese (≧25.0 kg/m2)	31 (88.6)	4 (11.4)		25 (71.4)	10 (28.6)		22 (62.9)	13 (37.1)	
**Living with family**									
No	480 (96.6)	17 (3.4)	.053	438 (88.1)	59 (11.9)	.662	400 (80.5)	97 (19.5)	.264
Yes	659 (94.1)	41 (5.9)		611 (87.3)	89 (12.7)		581 (83.0)	119 (17.0)	

(Continued)

**Relatives or friends who have been infected with SARS-CoV-2**									
No	896 (94.4)	53 (5.6)	.019	826 (87.0)	123 (13.0)	.220	779 (82.1)	170 (17.9)	.817
Yes	243 (98.0)	5 (2.0)		223 (89.9)	25 (10.1)		202 (81.5)	46 (18.5)	
**Smoking status**									
No	1025 (95.2)	52 (4.8)	.833	944 (87.7)	133 (12.3)	.770	887 (82.4)	190 (17.6)	.367
Yes	109 (95.6)	5 (4.4)		101 (88.6)	13 (11.4)		90 (78.9)	24 (21.1)	
**Change in alcohol consumption since start of pandemic**									
Decreased	231 (96.7)	8 (3.3)	.510	207 (86.6)	32 (13.4)	.563	196 (82.0)	43 (18.0)	.751
Unchanged or no alcohol consumption	848 (94.9)	46 (5.1)		785 (87.8)	109 (12.2)		735 (82.2)	159 (17.8)	
Increased	57 (95.0)	3 (5.0)		55 (91.7)	5 (8.3)		47 (78.3)	13 (21.7)	
**Change in financial situation since start of pandemic**									
Became difficult	106 (90.6)	11 (9.4)	.061	81 (69.2)	36 (30.8)	< .001	83 (70.9)	34 (29.1)	.001
Became slightly difficult	323 (94.4)	19 (5.6)		296 (86.5)	46 (13.5)		269 (78.7)	73 (21.3)	
Remained the same	626 (96.2)	25 (3.8)		595 (91.4)	56 (8.6)		553 (84.9)	98 (15.1)	
Became comfortable or became a bit comfortable	82 (96.5)	3 (3.5)		76 (89.4)	9 (10.6)		74 (87.1)	11 (12.9)	
**Physical activity level** [b]									
Low	610 (95.6)	28 (4.4)	.465	554 (86.8)	84 (13.2)	.297	518 (81.2)	120 (18.8)	.764
Moderate	383 (94.1)	24 (5.9)		356 (87.5)	51 (12.5)		337 (82.8)	70 (17.2)	
High	146 (96.1)	6 (3.9)		139 (91.4)	13 (8.6)		126 (82.9)	26 (17.1)	

(Continued)

**Change in physical activity level since start of pandemic**									
Decreased	679 (95.0)	36 (5.0)	.263	608 (85.0)	107 (15.0)	.002	568 (79.4)	147 (20.6)	.017
Unchanged or no exercise	371 (94.9)	20 (5.1)		358 (91.6)	33 (8.4)		333 (85.2)	58 (14.8)	
Increased	85 (98.8)	1 (1.2)		80 (93.0)	6 (7.0)		76 (88.4)	10 (11.6)	
**Life satisfaction** [c] (n, 1176)									
Median (IQR)	5 (3.0-7.0)	3 (2.0–5.0)	< .001	5 (3.0–7.0)	4 (3.0–6.0)	< .001	5 (3.0–7.0)	4 (3.0–6.0)	< .001
**Change in life satisfaction since start of pandemic**									
A lot worse	137 (87.8)	19 (12.2)	< .001	116 (74.4)	40 (25.6)	< .001	103 (66.0)	53 (34.0)	< .001
Worse	693 (96.3)	27 (3.8)		641 (89.0)	79 (11.0)		606 (84.2)	114 (15.8)	
Pretty much the same	242 (95.7)	11 (4.3)		229 (90.5)	24 (9.5)		215 (85.0)	38 (15.0)	
A lot better or better	67 (98.5)	1 (1.5)		63 (92.6)	5 (7.4)		57 (83.8)	11 (16.2)	
**Fear of COVID-19** [d] (n, 1196)									
Median (IQR)	17 (14.0–21.0)	20 (15.0–25.0)	< .001	17 (14.0–21.0)	20 (14.0–23.0)	< .001	17 (13.0–21.0)	19 (14.0–23.0)	.001

Abbreviations: BMI, Body Mass Index; COVID-19, Coronavirus Disease 2019; GAD-7, Generalized Anxiety Disorder-7; PHQ-9, Patient Health Questionnaire-9; ISI-7, Insomnia Severity Index-7; IQR, Interquartile Range

[a] For categorical variables, Fisher's exact test was used for the analysis of anxiety symptoms and sex; all other categorical variables were analyzed using the chi-square test, and continuous variables were analyzed using the Mann-Whitney U test

[b] Current level of physical activity was assessed with the International Physical Activity Questionnaire-Short Form

[c] Current life satisfaction was assessed using an 11-point Likert scale ranging from "extremely unsatisfied" to "extremely satisfied"

[d] Fear of COVID-19 was assessed with the Fear of Coronavirus-19 Scale
