# Supplementary material for: Impact of the COVID-19 pandemic on the mental health of nursing students in Japan: a cross-sectional study
Source: Environ Health Prev Med. 2022 Oct 15;27:40. doi: 10.1265/ehpm.22-00128 (PMC9640737; doi:10.1265/ehpm.22-00128)

Additional File 2. Coronavirus Disease 2019 (COVID-19) pandemic in Japan [a]

[a] In Japan, new confirmed cases of COVID-19 have been identified since January 2020, and the number of newly confirmed cases per day reached a record high of 26,050 on August 26, 2021. This survey was conducted from August 16, 2021 to October 16, 2021. The figure was created from open data on the Our World in Data website (https://ourworldindata.org/).

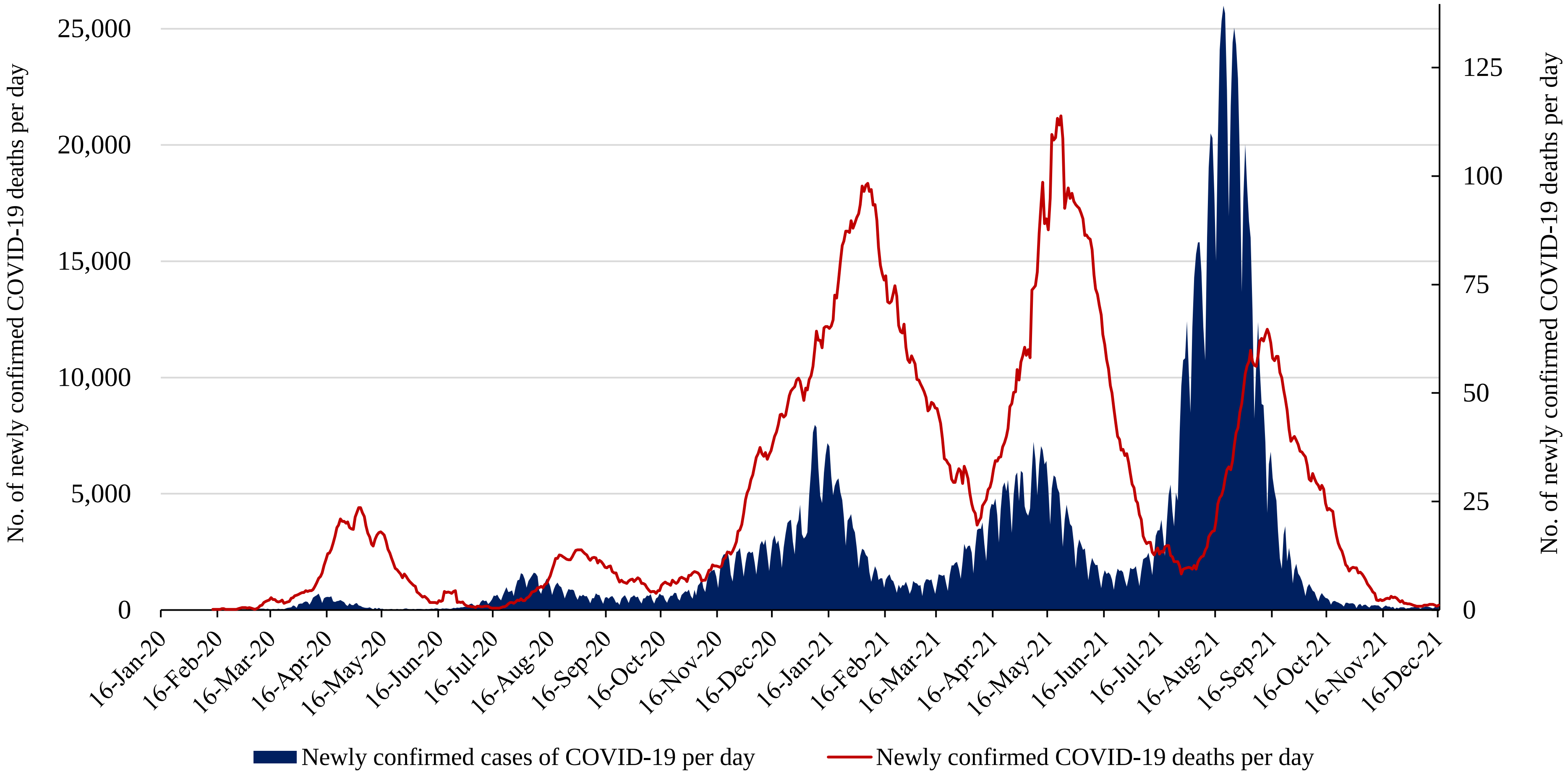

Supplement: Supplementary file 2 — Additional file 2: Coronavirus Disease 2019 (COVID-19) pandemic in Japana. [file ehpm-27-040-s002.pdf]
